# Effect of Extended Release Steroid Implants on the Contralateral Eye

**DOI:** 10.1186/s12886-022-02357-3

**Published:** 2022-03-22

**Authors:** Efrat Fleissig, Douglas Kenneth Sigford

**Affiliations:** 1 Department of Ophthalmology and Visual Sciences, University of Louisville School of Medicine, Louisville, Kentucky United States of America; 2 Department of Ophthalmology, Sackler Faculty of Medicine, Tel Aviv Medical Center, Tel-Aviv University, Tel-Aviv, Israel

**Keywords:** CST, Central subfield thickness, Macular Volume, Intravitreal extended release steroid injection, Contralateral eye, Dexamethasone intravitreal implant (Ozurdex), Fluocinolone acetonide intravitreal implant (ILUVIEN®)

## Abstract

**Purpose:**

To investigate the contralateral effect of extended release steroid implants on cystoid macular edema (CME).

**Methods:**

Retrospective study of patients with bilateral CME receiving intravitreal injections of long-acting intravitreal corticosteroid implants in one eye. Changes in CME and central subfield thickness (CST) in the contralateral eye on optical coherence tomography (OCT) were compared to an untreated control group. The main outcome measures were the change in central subfield thickness (CST) and the change in the macular volume.

**Results:**

Thirteen study patients and 14 controls were included in the study. There was no difference in the baseline LogMAR visual acuity (0.32 ± 0.35 vs 0.43 ± 0.26, *p* = 0.37) or the baseline central subfield thickness (341.4 ± 76.6 vs 296.5 ± 65.0 µm, *p* = 0.12) between groups. In the treatment group CST remained stable in 92.3% of the patients. Of the controls, CST worsened in 21.4% and remained stable in 78.6%. The mean change in CST (6.3 ± 30.3 vs. 27.5 ± 66.1 µm, *p* = 0.2) and the mean change in macular volume (0.08 ± 0.34 vs. -0.05 ± 0.21 mm^3^, *P* = 0.8) were not statistically different between the treatment group and control group. In the post-hoc analysis restricting the treatment group to patients who had not received intravitreal injections in the study eye within 6 months, CST decrement was not statistically significant (*p* = 0.11).

**Conclusion:**

In this study there was no statistically significant effect on CME of contralateral intravitreal corticosteroid implants.

## Introduction

Cystoid Macular Edema (CME) is an abnormal increase in fluid volume within the macula [1–3]. This process can result in symptomatic changes in vision. Various retinal conditions may lead to CME with a shared pathogenesis consisting of vascular hyperpermeability, leukostasis, and inflammation. The inflammatory process increases the vascular permeability through enhanced migration of immune cells followed by breakdown of the blood–retinal barrier (BRB) [3]. Inflammatory cytokines and angiogenic growth factors also contribute to the impairment of BRB and the increase in vascular permeability [3]. Common causes of CME include diabetic retinopathy, retinal vein occlusion, post-operative states, and uveitis [1–3]. Treatment options for CME vary and include anti-vascular endothelial growth factor (VEGF) injections, corticosteroid injections, extended release corticosteroids, topical steroid and nonsteroidal anti-inflammatory drops and carbonic anhydrase inhibitors [4, 5]. In uveitis, CME treatment may also include the use of immunomodulators [4]. Among the treatment options for chronic CME are two sustained release corticosteroid intravitreal implants. Dexamethasone intravitreal implant (Ozurdex®; Allergan, Inc., Irvine, CA) is one of the corticosteroids available for intravitreal use. It is injected in the form of a biodegradable implant that slowly releases 0.7 mg of active drug into the vitreous over a period of about 6 months. Dexamethasone intravitreal implant is approved by the Unites States Food and Drug Administration (FDA) in the treatment of patients with diabetic macular edema (DME), macular edema following retinal vein occlusion (RVO), and non-infectious posterior uveitis [6–8]. Fluocinolone acetonide intravitreal implant (ILUVIEN®; Alimera Sciences Inc., Alpharetta, GA) is a non-bioerodible insert containing 0.19 mg Fluocinolone acetonide in a 36-month sustained-release drug system. Iluvien® is FDA approved in the treatment of chronic DME in patients who have been previously treated with a course of corticosteroids with no significant rise in intraocular pressure [9]. Case reports indicate that the injection of dexamethasone intravitreal implant in one eye may result in CME reduction and improved inflammatory response in the contralateral eye, a phenomenon also seen with the intraocular injection of other medications [10–14]. In the clinical setting, bilateral injections may mask such an effect on the contralateral eye making this phenomenon difficult to study. In this retrospective study, our aim was to study the effect of extended release steroids on CME in the contralateral eye. To the best of our knowledge, this is the first study to assess this effect outside of case studies.

## Methods

This retrospective observational study was conducted at the University of Louisville after approval by the University of Louisville Institutional Review Board. The study adhered to the tenets of the Declaration of Helsinki and complied with Health Insurance Portability and Accountability Act guidelines. Informed consent to participate in the study was waived by the University of Louisville Institutional Review Board. The medical charts of all consecutive patients who underwent intravitreal injection with dexamethasone intravitreal implant (Ozurdex®) and fluocinolone acetonide intravitreal implant (Iluvien®) from January 1, 2015 to May 4, 2019 were retrospectively reviewed.

Subjects were included if they had bilateral macular edema secondary to diabetes, retinal vein occlusion, or uveitis, but were only undergoing treatment in one eye at the time of the measurements. The study eye, which was the contralateral untreated eye, was observed while the treatment eye continued to receive intravitreal injections with either corticosteroid implant. A control group of patients with macular edema, but without any recent treatments in either eye, was included for comparison. All subjects underwent bilateral spectral domain optical coherence tomography (OCT) examinations using the Zeiss OCT-AngioPlex (Cirrus HD-OCT 5000, Zeiss Meditec. Inc.) or the Heidelberg Spectralis SD-OCT (Heidelberg Engineering, Heidelberg, Germany) at baseline (day of injection) and at routine follow-up intervals. In order to create a washout period, exclusion criteria were intravitreal injection of the study eye with anti-VEGF agents within 2 months of the observation period, intravitreal injection of the study eye with fluocinolone intravitreal implant at any point, and intravitreal injection of the study eye with any other corticosteroid within 3 months of the observation period. Additional exclusion criteria included PRP in the study eye within 1 month, focal laser treatment, follow up on different OCT machines, and inadequate OCT images. Controls were excluded if they had any intravitreal injections in either eye within 3 months of the observation period.

The main outcome measures were the change in central subfield thickness (CST) and the change in the total macular volume of the central 6 mm ETDRS circle in the study (uninjected) eye of injected patients vs. controls. Improvement was regarded as decrease in the CST by 10% or more, and worsening was regarded as an increase in the CST by 10% or more of the central CST. A post-hoc analysis was also performed with a broader washout period where eyes with any intravitreal injections in the study eye of the treatment group within 6 months of the observation period were excluded.

Statistical analysis was carried out in R (The R Project for Statistical Computing, Vienna, Austria). Unpaired Student’s t-test was used to compare baseline characteristics. Paired Student’s t-test was used to compare the initial vs. final vision, CST, and macular volume within each group. Two-way ANCOVA was used to compare changes in OCT characteristics between groups with baseline measurements as covariates.

## Results

The baseline characteristics and main outcomes for treatment group and control group are presented in Table 1 and 2 respectively. Prior to the observation period, 10 of 13 eyes in the treatment group had received intravitreal injections in the study eye with a mean interval since last injection of 10.5 ± 16.5 months. The most recent intravitreal injection was bevacizumab in 7 patients, aflibercept in 2 patients, and dexamethasone intravitreal implant in 1 patient. Similarly, 7 of 14 eyes in the control group had received intravitreal injections in the study eye with a mean interval since last injection of 17.3 ± 10.4 months. The most recent intravitreal injection was bevacizumab in 4 patients, aflibercept in 2 patients, and triamcinolone acetonide in 1 patient.

**Table 1:** Baseline characteristics and outcomes of study patients

Age	Gender	Eye	Drug	Indication	Initial Visit			Final Visit			CST ∆ %	Volume ∆%	Drug (CE)	Interval To Last Injection (CE, months)
					Vision (Snellen)	CST(μm)	Volume (mm3)	Vision(Snellen)	CST(μm)	Volume(mm3)				
73	M	OD	Ozurdex	DME	20/25	290	10.4	20/25	315	10.58	8.62	1.73	Avastin	3
74	F	OS	Ozurdex	DME	20/40	282	9.01	20/30	288	9.11	2.13	1.11	Eylea	4
46	M	OS	Ozurdex	uveitis	20/25	372	10.28	20/25	383	10.42	2.96	1.36	Avastin	2
59	M	OS	Ozurdex	DME	20/400	439	10	20/500	533	10.88	21.41	8.8	Avastin	3.5
66	F	OD	Ozurdex	BRVO	20/50	494	10.52	20/50	499	10.98	1.01	4.37	Eylea	3
60	F	OD	iluvien	DME	20/50	321	8.27	20/60	295	8.45	-8.1	2.17	Ozurdex	3.5
54	F	OD	Ozurdex	CRVO, DME	20/25	306	9.14	20/25	322	9.38	5.23	2.63	Avastin	4
60	M	OS	Ozurdex	DME	20/20	337	8.95	20/20	344	8.76	2.08	-2.12	N/A	N/A
68	F	OS	Ozurdex	DME	20/80	243	8.15	20/100	222	7.6	-8.64	-6.74	N/A	N/A
71	M	OD	Ozurdex	DME	20/25	222	7.26	20/25	222	7.26	0	0	N/A	N/A
62	M	OS	iluvien	DME	20/20	380	9.18	20/20	373	9	-1.84	-1.96	Avastin	14.5
73	M	OS	Ozurdex	Pseudophakic CME	20/30	353	8.48	20/30	336	8.5	-4.82	0.23	Avastin	56
58	M	OS	Ozurdex	DME	20/60	399	9.92	20/100	388	9.7	-2.76	-2.21	Avastin	11

*CE* contralateral eye

**Table 2:** Baseline characteristics and outcomes of control patients

Age	Gender	Eye	Drug	Indication	Initial Visit			Final Visit			CST ∆ %	Volume ∆%	Drug (CE)	Interval To Last Injection (CE, months)
					Vision (Snellen)	CST (μm)	Volume(mm3)	Vision(Snellen)	CST(μm)	Volume (mm^3^)				
54	M	OS	N/A	DME	20/25	307	8.01	20/25	315	7.97	2.61	-0.48	Avastin	24
68	F	OS	N/A	DME	20/40	226	6.33	20/40	223	6.35	-1.33	0.23	Eylea	24
56	F	OS	Avastin	DME/RVO	20/25	301	7.49	20/25	301	7.46	0.00	-0.39	Avastin	9
58	M	OD	Kenalog	DME	20/50	396	7.69	20/50	399	7.80	0.76	1.47	Avastin	12
88	F	OD	N/A	BRVO	20/70	213	7.50	20/70	206	7.39	-3.29	-1.45	Eylea	36
72	M	OS	Avastin	DME	20/60	245	5.95	20/60	244	5.97	-0.41	0.32	Avastin	18
79	F	OD	N/A	BRVO	20/120	249	6.61	20/120	241	6.49	-3.21	-1.7	N/A	24
63	F	OS	Eylea	DME/BRVO	20/60	222	5.59	20/60	221	5.57	-0.45	-0.38	Eylea	16
67	F	OS	N/A	BVO	20/50	319	7.90	20/50	329	7.64	3.13	-3.4	N/A	36
96	F	OD	Avastin	BVO	20/120	298	6.77	20/120	290	6.76	-2.68	-0.14	N/A	36
71	M	OD	N/A	DME	20/50	389	6.28	20/40	385	6.17	-1.03	-1.69	Avastin	N/A
84	M	OD	N/A	BRVO	20/20	255	6.72	20/25	461	6.69	80.78	-0.46	Avastin	N/A
27	M	OS	N/A	DME	20/50	333	8.17	20/50	485	7.58	45.65	-7.26	N/A	N/A
72	F	OS	Eylea	DME	20/120	398	8.75	20/100	437	9.18	9.80	4.93	Avastin	6

There was no difference in the baseline LogMAR visual acuity (0.32 ± 0.35 vs 0.43 ± 0.26, *p* = 0.37) or the baseline central subfield thickness (341.4 ± 76.6 vs 296.5 ± 65.0 µm, *p* = 0.12) between groups. The baseline macular volume within the 6 mm central ETDRS circle was significantly higher in the treatment group (9.20 ± 0.99 vs 7.13 ± 0.94 mm^3^, *p* < 0.001). In the treatment group, 11 patients were treated with dexamethasone intravitreal implant and 2 patients were treated with fluocinolone acetonide intravitreal implant. The mean follow-up interval between OCT measurements of the study eye in this group was 5.5 ± 1.3 weeks. The mean time between OCT measurements in the control group was 7.7 ± 3.3 weeks.

The CST of the study eye in the treatment group changed from 341.4 ± 76.6 µm to 347.7 ± 91.5 µm (*p* = 0.47). CST remained stable as previously defined in 12 of the 13 (92.3%) patients in the treatment group. In the control group CST changed from 296.5 ± 65.0 µm to 324.1 ± 94.7 µm (*p* = 0.14). CST worsened in 21.4% (3 of 14 patients) and remained stable in 78.6% (11 of 14 patients) of the control group. In the treatment group, macular volume of the study eye changed from 9.20 ± 0.99 mm^3^ to 9.28 ± 1.20 mm^3^ (*p* = 0.42). In the control group, macular volume changed from 7.13 ± 0.94 mm^3^ to 7.07 ± 0.96 mm^3^ (*p* = 0.38). There was no statistically significant difference in either the change in CST (*p* = 0.24) or macular volume (*p* = 0.82) between the treatment and control groups. The mean logMAR visual acuity did not change in the study group (0.32 ± 0.35 vs. 0.36 ± 0.39, *p* = 0.13) or in the control group (0.43 ± 0.26 vs. 0.42 ± 0.24, *p* = 0.58).

**Fig. 1:**
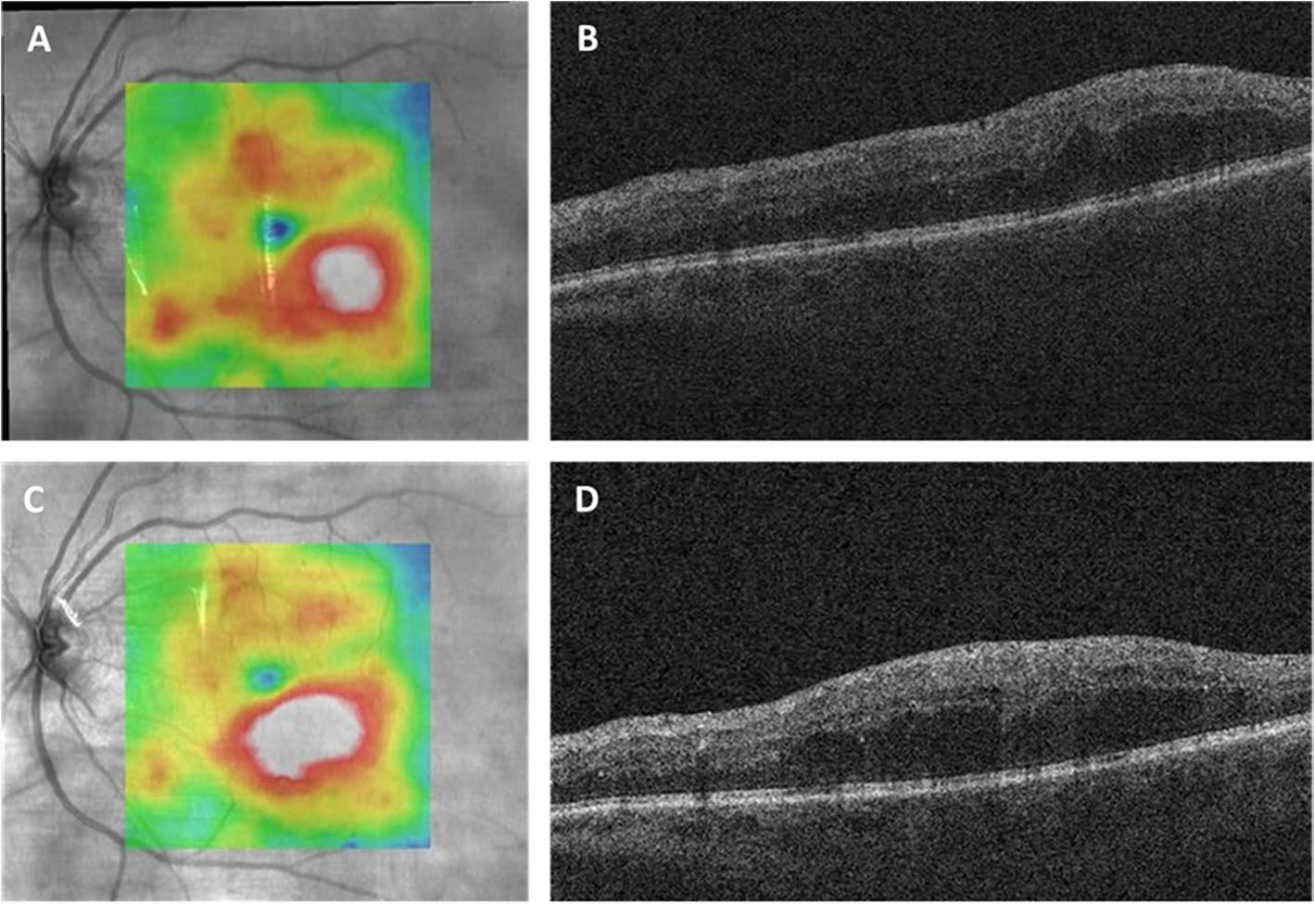
A 75-year-old diabetic patient received dexamethasone intravitreal implant in his right eye. Worsening of his extrafoveal edema of the left eye following this contralateral injection is shown by comparing the baseline optical coherence tomography (OCT) (**A** and **B**) to the follow-up OCT, 5 weeks post-injection (**C **and **D**). This change did not reach the pre-specified 10% cutoff for worsening in central subfield thickness

**Fig. 2:**
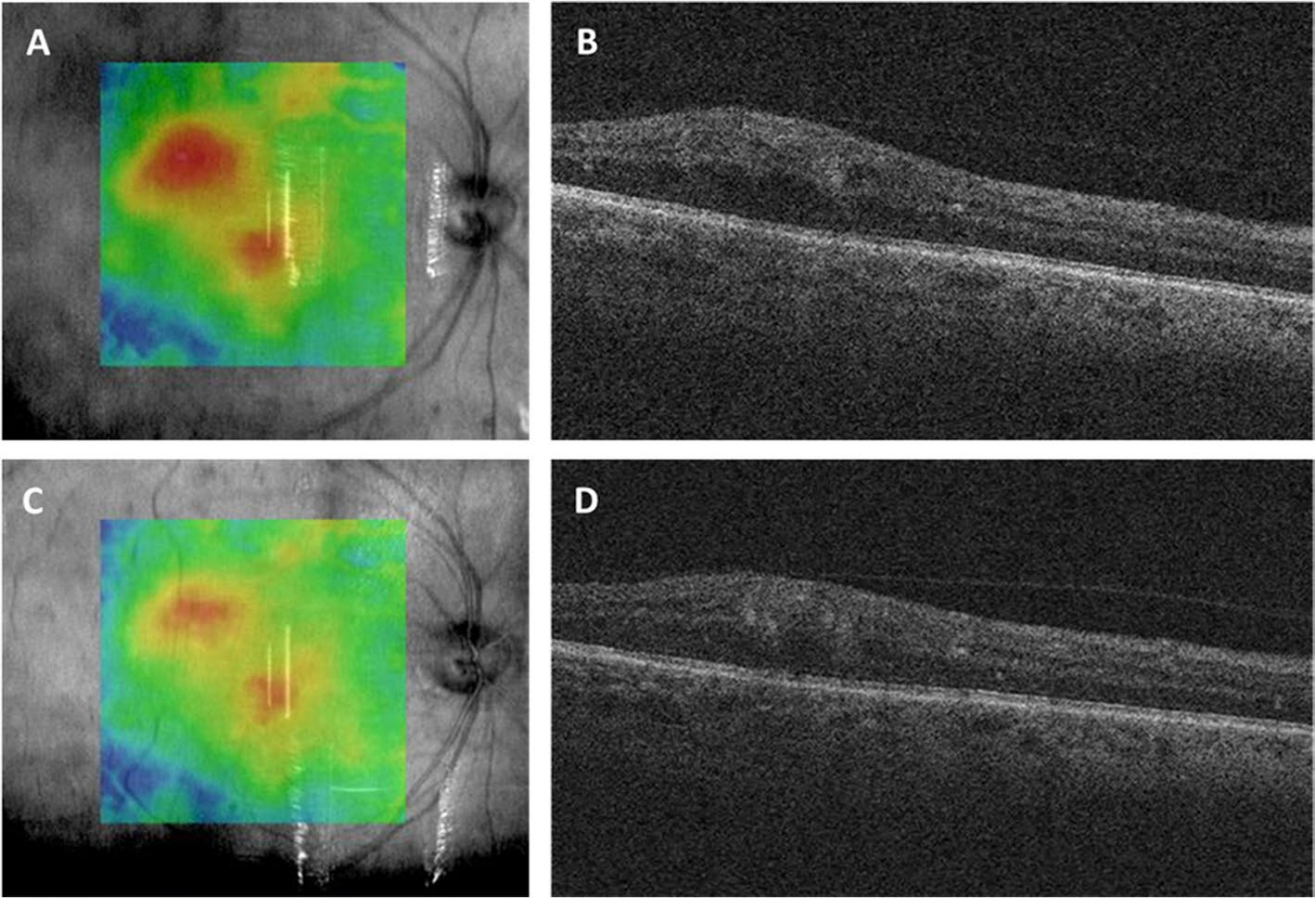
A 59-year-old diabetic patient received dexamethasone intravitreal implant in his left eye. Slight improvement of his extrafoveal edema of the right eye following this contralateral injection is shown by comparing the baseline optical coherence tomography (OCT) (**A** and **B**) to the follow-up OCT, 5 weeks post-injection (**C** and **D**). This change did not reach the pre-specified 10% cutoff for improvement in central subfield thickness

In the post-hoc analysis restricting the treatment group to patients who had not received intravitreal injections in the study eye within 6 months of the observation period, there were 6 qualifying eyes in the study group. CST decreased from 322.3 ± 73.1 µm to 314.2 ± 73.8 µm, which was not statistically significant (*p* = 0.11). The macular volume similarly decreased from 8.67 ± 0.92 mm^3^ to 8.47 ± 0.91 mm^3^, which was also not statistically significant (*p* = 0.08). Examples of patients with changes in their OCT parameters are shown in Figs. 1 and 2. Just as with the full cohort of patients, there was no statistically significant difference in either the change in CST (*p* = 0.2) or macular volume (*p* = 0.4) between the treatment and control groups in this subcategory.

## Discussion

In this small retrospective study, we did not find a consistent or statistically significant reduction in CME of fellow eyes of patients with bilateral CME receiving only unilateral extended release corticosteroid intravitreal implants. Most patients enrolled in the study received the dexamethasone intravitreal implant; however, two patients received the fluocinolone acetonide intravitreal implant.

There are case reports suggesting that the injection of dexamethasone intravitreal implant in one eye may result in effects in the other contralateral eye, a phenomenon seen with the intraocular injection of other medications [10–14]. Additionally, the pharmacokinetics of the dexamethasone implant support a possible bilateral effect as the drug can be detected at a low concentration in plasma for up to 90 days [15]. Indeed, we found that some study eyes showed a reduction in CST and macular volume after the contralateral eye received an intravitreal corticosteroid implant; however, this improvement was minimal and did not reach an arbitrary cutoff of 10% reduction in CST.

Patients with macular edema may show fluctuations in their level of CME, both improvement and worsening, spontaneously over time. Looking at the sham treatment group of several studies, both CST and visual acuity in DME and RVO may improve in up to 18% of patients [9, 16, 17]. For this reason, we included a control group in the present study. We did not find a statistically significant difference in the changes to either CST or macular volume between the treatment and control groups. The control group has a lower baseline macular volume than the treatment group. However, this is unlikely to have affected our results as it creates a ceiling effect for the controls which would actually increase the chance of finding a statistically significant result, not reduce that chance.

To the best of our knowledge, there are no large studies that compare the effect of corticosteroid implants in fellow eyes. Currie et. al. compared the injected and fellow eyes of patients receiving the fluocinolone acetonide intravitreal implant unilaterally for DME [18]. However, the fellow eyes were eligible to receive other intravitreal therapeutics, in contrast to this study. Nonetheless, there was only a minimal reduction of 3 µm in the central foveal thickness at 3 months and a 13 µm reduction at 1 year in the fellow eyes. These results fall within a similar range to our results, which showed a 6.3 µm increase in CST in the full cohort and an 8.1 µm reduction in CST in the cohort with the extended washout period. The pharmacokinetics of the fluocinolone acetonide intravitreal implant also would predict little to no effect on the contralateral eye as systemic detection is below the lower limit of quantitation in plasma (200 pg/mL) [19].

It is possible that we did not see an effect of these corticosteroids in the fellow eye due to an inadequate washout period. The period chosen for this study was selected as a balance between the duration of action of each medication and the desire for a larger cohort of patients. It is possible that an increase in CME from ongoing washout masked the positive effect of some contralateral injections. We explored this through a post-hoc analysis with an extended washout period. While both CST and macular volume fared slightly better in this cohort, the mean reduction in CST (8.1 µm) and macular volume (0.18 mm^3^) were both quite modest and not statistically significantly different from the control group. Another point that should be taken into consideration is that the some of the patients in the control group, may have reached a stable state, and therefore, may be masking a possible positive effect of the steroid preventing worsening of the edema in the study eye.

It is also possible we did not see an effect for other reasons. First, our patients had a relatively small amounts of CME at the beginning of the observation period with a mean starting CST in the study eye of 341 µm. However, some patients had CME away from the foveal center, and therefore macular volume was also included in the analysis. Limiting the study to patients with a larger CST would have further limited the number of patients as most patients with large amounts of CME undergo treatment. In the present study, even the patients with larger CST values did not show any substantial reduction in their CST values. It remains a possibility that some patients’ conditions may not have been responsive to corticosteroids, however this is less likely. One should also note that systemic absorption of intravitreal medications may vary from patient to patient or cohort to cohort. This may be altered by various effects such as disruption of the blood-retinal barrier, which is seen in diabetes, uveitis, and RVO [1–4, 20–22] and was hypothesized to be a factor in the bilateral effect of unilateral intravitreal dexamethasone implant in at least one case report [12]. It is difficult to control for the degree of blood-retinal barrier disruption and it remains possible that patients with substantial compromise may show differing effects of corticosteroids in a fellow eye. Lastly, this cohort included a small study group.

The limitations of our study have been touched upon and include a small study group, retrospective design, and a modest initial degree of CME. As the study and control groups are small, this study lacks the power to conclude no effect of intravitreal steroid injections on the contralateral eye. However, this is a very unique and limited subgroup of patients who have bilateral edema with treatment in only one eye. Despite these limitations, this study remains valuable, having a larger collection of such patients than has been previously published and including a control group.

## Conclusions

In conclusion, we found that extended release intravitreal corticosteroids did not have a statistically significant effect on the CME of contralateral eyes. While a fellow eye response may occur in some patients, as is evidenced by prior case reports, the effect should not be expected routinely and there are several factors that may be more important in the evolution of CME. Larger scale prospective studies are needed to better characterize this phenomenon.

## Data Availability

All data generated or analyzed during this study are included in this published article.

## References

[1] Coscas G: Macular Edema. 2nd, revised and extended edition. Dev Ophthalmol. Basel, Karger, 2017(58);1–10 10.1159/000455264. 10.1159/000455264 28351040

[2] McCullough PC, Koester CJ, Campbell CJ, Anderson EA (1983). An evaluation of the clinical role of vitreous fluorophotometry. Trans Am Ophthalmol Soc.

[3] Chung YR, Kim YH, Lee SY, Byeon HE, Lee K (2019). Insights into the pathogenesis of cystoid macular edema: leukostasis and related cytokines. Int J Ophthalmol.

[4] Sacconi R, Giuffrè C, Corbelli E, Borrelli E, et al. Emerging therapies in the management of macular edema: a review. F1000Res. 2019;8:F1000 Faculty Rev-1413. 10.12688/f1000research.19198.1 10.12688/f1000research.19198.1 PMC6694451 31448093

[5] Zur D, Loewenstein A (2017). Postsurgical Cystoid Macular Edema. Dev Ophthalmol.

[6] Lowder C, Belfort R, Lightman S, Foster CS (2011). Dexamethasone intravitreal implant for noninfectious intermediate or posterior uveitis. Arch Ophthalmol..

[7] Haller JA, Bandello F, Belfort R, Blumenkranz MS (2011). Dexamethasone intravitreal implant in patients with macular edema related to branch or central retinal vein occlusion twelve-month study results. Ophthalmology.

[8] Boyer DS, Yoon YH, Belfort R, Bandello F (2014). Three-year, randomized, sham-controlled trial of dexamethasone intravitreal implant in patients with diabetic macular edema. Ophthalmology.

[9] Campochiaro PA, Brown DM, Pearson A, Ciulla T (2012). Sustained delivery fluocinolone acetonide vitreous inserts provide benefit for at least 3 years in patients with diabetic macular edema. Ophthalmology.

[10] Sharma A, Sheth J, Madhusudan RJ, Sundaramoorthy SK (2013). Effect of intravitreal dexamethasone implant on the contralateral eye: a case report. Retin Cases Brief Rep.

[11] Ciszewska J, Brydak-Godowska J, Kuźnik-Borkowska A, Kęcik D (2016). The effect of a single intravitreal implantation of dexamethasone on the contralateraleye in bilateral non-infectious uveitis - case report. Eur Rev Med Pharmacol Sci.

[12] Habot-Wilner Z, Sorkin N, Goldenberg D, Goldstein M (2015). Bilateral effect of unilateral dexamethasone intravitreal implant in a case of noninfectious uveitic macular edema and vitritis. Retin Cases Brief Rep.

[13] Al-Dhibi H, Khan AO (2009). Bilateral response following unilateral intravitreal bevacizumab injection in a child with uveitic cystoidmacular edema. J AAPOS.

[14] Acharya NR, Sittivarakul W, Qian Y, Hong KC (2011). Bilateral effect of unilateral ranibizumab in patients with uveitis-related macular edema. Retina.

[15] Chang-Lin JE, Attar M, Acheampong AA, Robinson MR (2011). Pharmacokinetics and pharmacodynamics of a sustained-release dexamethasone intravitreal implant. Invest Ophthalmol Vis Sci.

[16] David S.Boyer, Young Hee Yoon, Rubens Belfort, Francesco Bandello,  (2014). Three-Year, Randomized, Sham-Controlled Trial of Dexamethasone Intravitreal Implant in Patients with Diabetic Macular Edema, Ophthalmology.

[17] Haller JA, Bandello F, Belfort R, Blumenkranz MS (2010). Randomized, sham-controlled trial of dexamethasone intravitreal implant in patients with macular edema due to retinal vein occlusion. Ophthalmology.

[18] Currie CJ, Holden SE, Berni E, Owens DR (2017). Evaluation of the clinical effectiveness of fluocinolone acetonide 190 µg intravitreal implant in diabetic macular edema: a comparison between study and fellow eyes. Curr Med Res Opin.

[19] Kane FE, Green KE (2015). Ocular pharmacokinetics of fluocinolone acetonide following Iluvien implantation in the vitreous humor of rabbits. J Ocul Pharmacol Ther.

[20] Chahal PS, Fallon TJ, Kohner EM (1986). Measurement of Blood-Retinal Barrier Function in Central Retinal Vein Occlusion. Arch Ophthalmol.

[21] José G Cunha-Vaz. Studies on the Pathophysiology of Diabetic Retinopathy: The Blood-Retinal Barrier in Diabetes. Diabetes 1983;32:20–27. 10.2337/diab.32.2.s20 6400666

[22] Klaassen I, Van Noorden CJ, Schlingemann RO (2013). Molecular basis of the inner blood-retinal barrier and its breakdown in diabetic macular edema and other pathological conditions. Prog Retin Eye Res.

